# Antimicrobial and Antioxidant Performance of Various Essential Oils and Natural Extracts and Their Incorporation into Biowaste Derived Poly(3-hydroxybutyrate-*co*-3-hydroxyvalerate) Layers Made from Electrospun Ultrathin Fibers

**DOI:** 10.3390/nano9020144

**Published:** 2019-01-23

**Authors:** Kelly J. Figueroa-Lopez, António A. Vicente, Maria A. M. Reis, Sergio Torres-Giner, Jose M. Lagaron

**Affiliations:** 1 Novel Materials and Nanotechnology Group, Institute of Agrochemistry and Food Technology (IATA), Spanish National Research Council (CSIC), Calle Catedrático Agustín Escardino Benlloch 7, Paterna, 46980 Valencia, Spain; kjfigueroal@iata.csic.es (K.J.F.-L.); storresginer@iata.csic.es (S.T.-G.); 2 Centre of Biological Engineering, University of Minho, Campus Gualtar, 4710-057 Braga, Portugal; avicente@deb.uminho.pt; 3 UCIBIO-REQUIMTE, Departamento de Química, Faculdade de Ciências e Tecnologia, Universidade Nova de Lisboa, Campus de Caparica, 2829-516 Caparica, Portugal; amr@fct.unl.pt

**Keywords:** PHBV, oregano, rosemary, green tea, electrospun nanofibers, antibacterial, antioxidant

## Abstract

In this research, the antibacterial and antioxidant properties of oregano essential oil (OEO), rosemary extract (RE), and green tea extract (GTE) were evaluated. These active substances were encapsulated into ultrathin fibers of poly(3-hydroxybutyrate-*co*-3-hydroxyvalerate) (PHBV) derived from fruit waste using solution electrospinning, and the resultant electrospun mats were annealed to produce continuous films. The incorporation of the active substances resulted in PHBV films with a relatively high contact transparency, but it also induced a slightly yellow appearance and increased the films opacity. Whereas OEO significantly reduced the onset of thermal degradation of PHBV, both the RE and GTE-containing PHBV films showed a thermal stability profile that was similar to the neat PHBV film. In any case, all the active PHBV films were stable up to approximately 200 °C. The incorporation of the active substances also resulted in a significant decrease in hydrophobicity. The antimicrobial and antioxidant activity of the films were finally evaluated in both open and closed systems for up to 15 days in order to anticipate the real packaging conditions. The results showed that the electrospun OEO-containing PHBV films presented the highest antimicrobial activity against two strains of food-borne bacteria, as well as the most significant antioxidant performance, ascribed to the films high content in carvacrol and thymol. Therefore, the PHBV films developed in this study presented high antimicrobial and antioxidant properties, and they can be applied as active layers to prolong the shelf life of the foods in biopackaging applications.

## 1. Introduction

The packaging industry requires the development of new plastic materials with active properties, based on the demand by consumers for safer and more nutritive food [1]. Moreover, the growing concern over the environmental problems caused by petroleum-derived materials has led to the search for new renewable raw materials for the development of compostable packaging [2,3]. Polyhydroxyalkanoates (PHAs) are amongst the most promising biopolymers, being a group of totally renewable, biodegradable, and biocompatible aliphatic polyesters. PHAs are synthesized in the cytoplasm of a wide range of bacteria from glucose-rich substrates [4,5]. Some PHAs, such as poly(3-hydroxybutyrate) (PHB), poly(3-hydroxybutyrate-*co*-3-hydroxyvalerate) (PHBV), poly(3-hydroxybutyrate-*co*-4-hydroxybutyrate) (P(3HB-*co*-4HB)), and poly(3-hydroxybutyrate-*co*-3-hydroxyhexanoate) (PHBH) are currently being employed to develop bioplastic packaging articles, such as injection-molded pieces, compression-molded sheets, and films [6,7,8,9].

Active packaging technology is mostly related to materials and articles that are intended to extend food shelf life, and also to improve packaged food conditions by interacting with the food product and/or with its internal packaging environment. Active packaging materials are usually designed to deliberately incorporate components, which would then release and/or absorb substances into or from the packaged food or the environment surrounding the food [10]. Active packaging systems can therefore extend the shelf life of food products and reduce food waste by maintaining the quality of food products for longer, increasing product safety by securing the foods against pathogens, and enhancing the convenience of food processing, distribution, retailing, and consumption [11]. Concerning the active packaging materials, these are classified as either active scavenging types (e.g., oxygen scavengers) [12] or active releasing types (e.g., antioxidants) [13]. Active releasing-type packaging can provide novel “extra” functions, such as aromatic, antioxidant, and long-term antimicrobial properties [14]. In particular, active-releasing antimicrobial packaging applications are directly related to food microbial safety, as well as to shelf life extension, by preventing the growth of spoilage and/or pathogenic microorganisms [15,16]. The growth of spoilage microorganisms can not only reduce the food shelf life, but it can also endanger public health (particularly in the case of pathogenic microorganisms).

Active properties can be conferred by the incorporation into the packaging materials of substances with inherent antioxidant and antimicrobial properties, such as essential oils (EOs) [17], natural extracts (NEs) [18], and/or inorganic and metal nanoparticles [19]. EOs are volatile compounds obtained from aromatic plants that produced them naturally as secondary metabolites [20]. EOs and NEs are mainly composed of terpenoids, phenolic, and aromatic compounds, and their composition can widely vary depending on the edaphoclimatic characteristics of the plant, the part of the plant (i.e., flower, seed, leaves, fruits, stems, and others), and the extraction procedure [21]. There is great interest in the use of these natural products because they are classified as generally recognized as safe (GRAS) food additives by the Food and Drug Administration (FDA) [22]. 

In line with this, over the last few years, different EOs and NEs have been proposed as alternative sources of antimicrobials in packaging materials. Within the great variety of EOs, oregano essential oil (OEO) from *Origanum vulgare* is well known for its antioxidative and antimicrobial activities [23]. The EO content in the oregano plant fluctuates from 0.5–2% [24] up to 7% [25]. Its main constituents are the isomer phenols, carvacrol and thymol, which represent up to 80% and 64%, respectively [26]. In addition, up to 52% of each of their precursor monoterpenes, p-cymene and γ-terpinene, as well as terpinen-4-ol, linalool, β-myrcene, trans-sabinene hydrate, and β-caryophyllene, are also present [27]. Rosemary extract (RE), which is obtained from *Rosmarinus officinalis*, is an aromatic plant belonging to the Lamiaceae family [28], and it also presents strong antimicrobial and antifungal properties [29]. The active properties of RE are primarily conferred by its phenolic, and the volatile constituents carnosol, carnosic acid, and rosmarinic acid [30]. Its minor components may have a potential influence on biological activity due to the possibility of synergistic effects amongst their components [31]. Finally, green tea tree extract (GTE) obtained from *Camellia sinensis* has gained significant attention in recent years. GTE is mainly composed of gallic acid, theobromine, chlorogenic acid, and caffeic acid [32]. In view of the potential uses of these natural products as effective antimicrobial and antioxidants for food preservation, they can be great candidates for incorporation into PHA films to generate active packaging articles.

Since most EOs and NEs are volatile compounds, they require the use of manufacturing methods that are carried out at room temperature to preserve their original properties. In this sense, the electrospinning technique is an emerging technology in the food packaging field [33,34], which is based on the application of electrostatic forces to polymer solutions to generate polymer fibers with diameters ranging from below 100 nm to several micrometers. Owing to the high surface-to-volume ratio of the electrospun fibers and the controllable pore size of the electrospun mats, several active and bioactive applications have been proposed in recent years [35], including the development of novel antimicrobial systems [36]. Since the electrospinning technique is frequently performed at room temperature, it facilitates the processing of thermolabile substances [37]. In addition, in a packaging application context, the ultrathin electrospun PHA fiber mats can be further converted into continuous films through the application of a thermal post-treatment below the polymer’s melting temperature (*T_m_*), i.e., the so-called annealing [38,39]. 

The objective of this research was to develop, for the first time, electrospun PHBV films containing OEO, RE, and GTE, in order to obtain active packaging layers with antioxidant and antimicrobial properties. Likewise, the morphological, optical, and thermal properties of the electrospun biopolymer films were also evaluated.

## 2. Materials and Methods 

### 2.1. Materials 

PHBV copolyester was produced at a pilot-plant scale at the Universidade NOVA de Lisboa (Lisboa, Portugal). This biopolymer was obtained using mixed microbial cultures fed with fermented fruit waste derived from the manufacturing of fruit juice, supplied by SumolCompal S.A. (Lisbon, Portugal). The molar fraction of the 3-hydroxyvalerate (HV) in the copolyester used was 20%. The synthesis, purification, and characterization details of this biopolymer was thoroughly described in Reference [39].

Chloroform, reagent grade with 99.8% purity, and methanol, HPLC grade with 99.9% purity, were purchased from Panreac S.A. (Barcelona, Spain). Additionally, 1-Butanol, reagent grade with 99.5% purity, and 2,2-diphenyl-1-picrylhydrazyl radical (DPPH) were purchased from Sigma Aldrich S.A. (Madrid, Spain).

OEO had a purity >99% and a relative density of 0.925–0.955 g/mL. RE presented a relative density of 0.915–0.926 g/mL, an acidity index of ≤1 mg KOH/g, an iodine index of 80.0–145.0%, a saponification index of 180–200 mg KOH/g, and a peroxide index of ≤5.0 meqO_2_/kg. GTE showed a relative density of 0.915–0.925 g/mL, an acidity index of ≤1 mg KOH/g, an iodine index of 80–145%, a saponification index of 188–195 mg KOH/g, and a peroxide index of ≤5.0 meq O_2_/Kg. All natural products were obtained from Gran Velada S.L. (Zaragoza, Spain) and were processed as received.

### 2.2. Preparation of the Solutions

The PHBV solution was prepared by dissolving 10% (wt./vol.) of biopolymer in a chloroform/1-butanol 75:25 (vol./vol.) mixture, both reagent grades, at room temperature. The OEO, RE, and GTE, were all added to the solution at 10 wt.% in relation to the PHBV and stirred for 24 h until a single-phase solution was obtained.

### 2.3. Characterization of the Solution Properties

All the PHBV solutions were analyzed in terms of their viscosity, surface tension, and conductivity. The apparent viscosity (ηa) was determined at 100 s^−1^ using a rotational viscosity meter Visco BasicPlus L from Fungilab S.A. (San Feliu de Llobregat, Spain). The surface tension was measured following the Wilhemy plate method using an EasyDyne K20 tensiometer from Krüss GmbH (Hamburg, Germany). The conductivity was evaluated using a conductivity meter HI9819X from Hanna Instruments (Woonsocket, Rhode Island, USA). All the measurements were carried out at room temperature and in triplicate. 

### 2.4. Electrospinning

The PHBV solutions containing OEO, RE, and GTE were each electrospun for 3 h onto an aluminum foil using a high-throughput electrospinning/electrospraying pilot line Fluidnatek^®^ LE-500 with temperature and relative humidity (RH) control, which was manufactured and commercialized by Bioinicia S.L. (Valencia, Spain). The solutions were then processed at 25 °C and 40% RH under a constant flow using a 24 emitter multi-nozzle injector, scanning vertically onto the metallic plate. A dual polarization added voltage of 38 kV, and a flow-rate of 4 mL/h per single emitter and a tip-to-collector distance of 20 cm were used. A neat PHBV solution was electrospun in identical conditions as the control.

Thereafter, an annealing treatment was applied to the resultant electrospun mats. This process was performed in a 4122-model press from Carver, Inc. (Wabash, IN, USA) at 125 °C, for 5 s, without pressure. The resultant film samples had an average thickness in the 60–80 µm range.

### 2.5. Characterization of the Electrospun Materials

#### 2.5.1. Film Thickness

Before testing, the thickness of the PHBV films containing the natural products was measured using a digital micrometer (S00014, Mitutoyo, Corp., Kawasaki, Japan) with ± 0.001 mm accuracy. Measurements were performed and averaged at five different points, one in each corner and one in the middle.

#### 2.5.2. Morphology 

The morphology of the electrospun PHBV fibers and their films containing the OEO, RE, and GTE were examined by scanning electron microscopy (SEM). The micrographs were taken using a Hitachi S-4800 electron microscope (Tokyo, Japan), at an accelerating voltage of 10 kV and a working distance of 8–10 mm. The samples were previously sputtered with a gold-palladium mixture for 3 min under vacuum. The average fiber diameter was determined via the ImageJ software v 1.41 using at least 20 SEM images.

#### 2.5.3. Transparency

The light transmission of the PHBV films was determined in specimens of 50 × 30 mm^2^ by quantifying the absorption of light at wavelengths between 200 and 700 nm, using an ultraviolet–visible (UV–Vis) spectrophotometer VIS3000 from Dinko Instruments (Barcelona, Spain). The transparency value (T) of the films was calculated using Equation (1) [1], whereas their opacity value (O) was determined using Equation (2) [40]:

(1) $$T=\frac{A_{600}}{L}$$

(2) $$O=A_{500}L$$

where $A_{500}\;\mathrm{and}\;A_{600}$ are the absorbance values at 500 and 600 nm, respectively, and *L* is the film thickness (mm).

#### 2.5.4. Color

The PHBV films color was determined using a chroma meter CR-400 (Konica Minolta, Tokyo, Japan). The color difference (ΔE*) was calculated using the following Equation (3) [1], as defined by the Commission Internationale de l’Eclairage (CIE):

(3) $$\Delta E^{*}={\left[{\left(\Delta L^{*}\right)}^{2}+{\left(\Delta a^{*}\right)}^{2}+{\left(\Delta b^{*}\right)}^{2}\right]}^{0.5}$$

where ΔE*, ΔL*, Δa*, and Δb* correspond to the differences between the color parameters of the sample films and the values of the control film (a* = 0.87, b* = –0.38, L* = 89.82).

#### 2.5.5. Thermal Analysis

Thermogravimetric analysis (TGA) of the neat OEO, RE, and GTE in their liquid form, and the PHBV films, was performed under a nitrogen atmosphere in a Thermobalance TG-STDA Mettler Toledo model TGA/STDA851e/LF/1600 analyzer (Greifensee, Switzerland). The TGA curves were obtained after conditioning the samples in the sensor for 5 min at 30 °C. The samples were then heated from 25 °C to 700 °C, at a heating rate of 10 °C/min. All tests were carried out in triplicate.

#### 2.5.6. Water Contact Angle Measurements

The PHBV films surface wettability was evaluated using dynamic water contact angle (WCA) measurements in an optical tensiometer (Theta Lite, Staffordshire, UK). Five droplets were seeded at 5 µL/s on the film surfaces of each studied material sizing of 2 × 5 cm^2^, in triplicate, and the resulting average contact angle was calculated.

#### 2.5.7. Antimicrobial Activity 

*Staphylococcus aureus* (*S. aureus*) CECT240 (ATCC 6538p) and *Escherichia coli* (*E. coli*) CECT434 (ATCC 25922) strains were obtained from the Spanish Type Culture Collection (CECT, Valencia, Spain) and stored in phosphate buffered saline (PBS), with 10 wt.% tryptic soy broth (TSB, Conda Laboratories, Madrid, Spain) and 10 wt.% glycerol (99.5% purity, Sigma Aldrich S.A. Madrid, Spain) at –80 °C. Prior to each study, a loopful of bacteria was transferred to 10 mL of TSB and incubated at 37 °C for 24 h. A 100 µL aliquot from the culture was again transferred to the TSB and grown at 37 °C to the mid-exponential phase of growth. The approximate count of 5 × 10^5^ CFU/mL of culture had an absorbance value of 0.20, as determined by the optical density at 600 nm (UV 4000 spectrophotometer, Dinko Instruments, Barcelona, Spain).

The minimum inhibitory concentration (MIC) and minimum bactericide concentration (MBC) of the OEO, RE, and GTE against the selected food-borne bacteria was tested following the plate micro-dilution protocol, as described in the Methods for Dilution Antimicrobial. Susceptibility Tests for Bacteria That Grow Aerobically; Approved Standard Tenth. Edition (M07-A10) by the Clinical and Laboratory Standards Institute (CLSI). For this, a 96-well plate with an alpha numeric coordination system (columns 12 and rows A-H) was used, where 10 µl of the tested samples were introduced into the wells with 90 µl of the bacteria medium. In the wells corresponding to A, B, C, E, F, and G columns, different concentrations of the natural products, that is, 0.312, 0.625, 1.25, 2.5, 5, 10, 20, 40, 80, 160 µl/ml, were tested, in triplicate, from rows 1 to 10. Columns D and H were used as control of the natural extracts in the TSB without bacteria. Row 11 was taken as a positive control, that is, only the TSB, and row 12 was used as a negative control, that is, *S. aureus* and *E. coli* in the TSB. The plates were incubated at 37 °C for 24 h. Thereafter, 10 µl of resazurin sodium salt (MP biologicals, Illkirch, France), a metabolic indicator, was added to each well and incubated again at 37 °C for 2 h. Upon obtaining the resazurin change, the wells were read through the color difference. The MIC and MBC values were determined as the lowest concentration of the natural products presenting bacteriostatic and bactericide effects, respectively [41].

The antimicrobial performance of the electrospun PHBV films was evaluated using a modification of the Japanese Industrial Standard (JIS) Z2801 (ISO 22196:2007) [42]. To this end, a microorganism suspension of *S. aureus* and *E. coli* was applied onto the test films, that is, containing the natural extracts, and the negative control film, that is, without the natural extracts, all sizing 1.5 × 1.5 cm^2^. Tests were performed in either hermetically closed or open vials with a volume of 20 mL. After incubation at 24 °C and at a relative humidity (RH) of, at least, 95% for 24 h, the bacteria were recovered with PBS, 10-fold serially diluted, and incubated at 37 °C for 24 h in order to quantify the number of viable bacteria by a conventional plate count. The antimicrobial activity was evaluated after 1 (initial day), 8, and 15 days. The value of the antimicrobial reduction (R) was calculated following Equation (4):

(4) $$R=\left[log\left(\frac{B}{A}\right)-log\left(\frac{C}{A}\right)\right]=log\left(\frac{B}{C}\right)$$

where *A* is the average of the number of viable bacteria on the control film sample immediately after inoculation, *B* is the average of the number of viable bacteria on the control film sample after 24 h, and *C* is the average of the number of viable bacteria on the test film sample after 24 h. Three replicate experiments were performed for each sample and the following assessment was conducted: Nonsignificant (R < 0.5), slight (R ≥ 0.5 and <1), significant (R ≥ 1 and <3), and strong (R ≥ 3) as in Reference [43].

#### 2.5.8. Antioxidant Activity

The DPPH inhibition assay was used to evaluate the free radical scavenging activity of the neat OEO, RE, GTE in their oil forms, in the electrospun PHBV fibers (at day 1) and their corresponding films (at 1, 8, and 15 days). Samples were weighed in triplicate in cap vials and then an aliquot of the DPPH solution (0.05 g/L in methanol) was added to each one. Vials without samples were also prepared as controls. All the samples were prepared and immediately stored at room temperature for 2 h in darkness. After this, the absorbance of the solution was measured at 517 nm in the UV 4000 spectrophotometer from Dinko Instruments. Results were expressed as the percentage of inhibition to DPPH following Equation (5) [44] and μg equivalent of trolox per gram of sample, employing a previously prepared calibration curve of trolox.

(5) $$Inhibition\;DPPH\;\left(\%\right)=\frac{A_{Control}-\left(A_{sample}-A_{blank}\right)}{A_{control}}*100$$

where $A_{control},\;A_{blank},\;\mathrm{and}\;A_{sample}$ are the absorbance values of the DPPH solution, methanol with the test sample, and the test sample, respectively.

#### 2.5.9. Statistical Analysis 

The solution properties, color, transparency, and opacity values, and contact angle values were evaluated through analysis of variance (ANOVA) using STATGRAPHICS Centurion XVI v 16.1.03 from StatPoint Technologies, Inc. (Warrenton, VA, USA). Fisher’s least significant difference (LSD) was used at the 95% confidence level (*p* < 0.05). Mean values and standard deviations were also calculated.

## 3. Results

### 3.1. Solution Properties 

The use of polymer solution with the adequate properties is a key parameter to obtain uniform fibers during electrospinning [45]. In Table 1, the viscosity, surface tension, and conductivity of the PHBV solutions containing OEO, RE, and GTE at 10 wt.% are shown. The neat PHBV solution, without OEO and NEs, showed the highest viscosity value, that is, 212.4 cP. This value was relatively similar to that reported by Melendez-Rodriguez et al. [39], who obtained a value of viscosity of 296.8 cP for a PHBV solution in 2,2,2-trifluoroethanol (TFE) at 2 wt%. This difference could be mainly ascribed to the solvent type and, more likely, to the use of a biopolymer with a higher molecular weight (*M_W_*). When OEO, RE, and GTE were added, the viscosity of the PHBV solution slightly decreased. This effect could be ascribed to a reduction of the molecular cohesion forces in the biopolymer due to the presence of the active substances. This result was in agreement with, for instance, previous research works reported by Arfa et al. [46] and Jouki et al. [47], showing that the addition of either OEO or its active components decreased the apparent viscosity of polymer solutions of mucilage and soy protein (SP). In any case, for all the here-prepared PHBV solutions, the viscosity values were within the range of values reported by other authors, that is, from 1 to 20 poise (P), for the formation of homogeneous fibers during electrospinning [48].

The surface tension and conductivity of the solutions showed no significant differences (*p* > 0.05). However, it is worthy to mention that other authors have observed changes in the latter parameters when homogenization treatments (e.g., ultrasound, sonication, etc.) were applied to polymer solutions containing different EOs and NEs [49,50]. Moreover, in the case of polymer emulsions, the incorporation of these natural extracts into the oil or water phases resulted in an increasing drop size that destabilized the emulsion [51]. Therefore, the similar values observed for the neat PHBV solution and the PHBV solutions containing the OEO, RE, and GTE suggested that a high homogenization was achieved in all cases. Therefore, it was considered that the resulting solutions presented the adequate values for being processed by electrospinning.

### 3.2. Morphology

**Figure 1:**
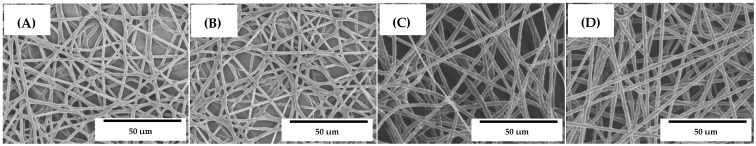
Scanning electron microscopy (SEM) micrographs of the electrospun fibers of: (**A**) Neat poly(3-hydroxybutyrate-*co*-3-hydroxyvalerate) (PHBV); (**B**) Oregano essential oil (OEO)-containing PHBV; (**C**) Rosemary extract (RE)-containing PHBV; (**D**) Green tea tree extract (GTE)-containing PHBV. Scale markers of 50 µm.

The morphology of the electrospun ultrathin neat PHBV fibers and the PHBV fibers containing the OEO, RE, and GTE was analyzed by SEM and the images are shown in Figure 1. The neat PHBV fibers, without EOs and NEs, were relatively uniform and presented a mean diameter of approximately 1 µm, as seen in Figure 1A. The morphology of the here-obtained electrospun ultrathin PHBV fibers was similar to those fibers reported by Melendez-Rodriguez et al [39], showing diameters of ~1.32 µm. The PHBV fibers containing 10 wt% OEO, RE, and GTE are presented in Figure 1B–D, respectively. The diameters of the fibers were relatively similar, with a mean size of approximately 0.8 μm. The reduction achieved in the fiber diameter could be related to the slightly lower viscosities observed for the PHBV solutions containing the active substances. It was also evident that all the electrospun fibers were uniform and smooth, without any superficial and structural defects, which indicated that the addition of both EOs and NEs did not alter the fiber formation during electrospinning.

**Figure 2:**
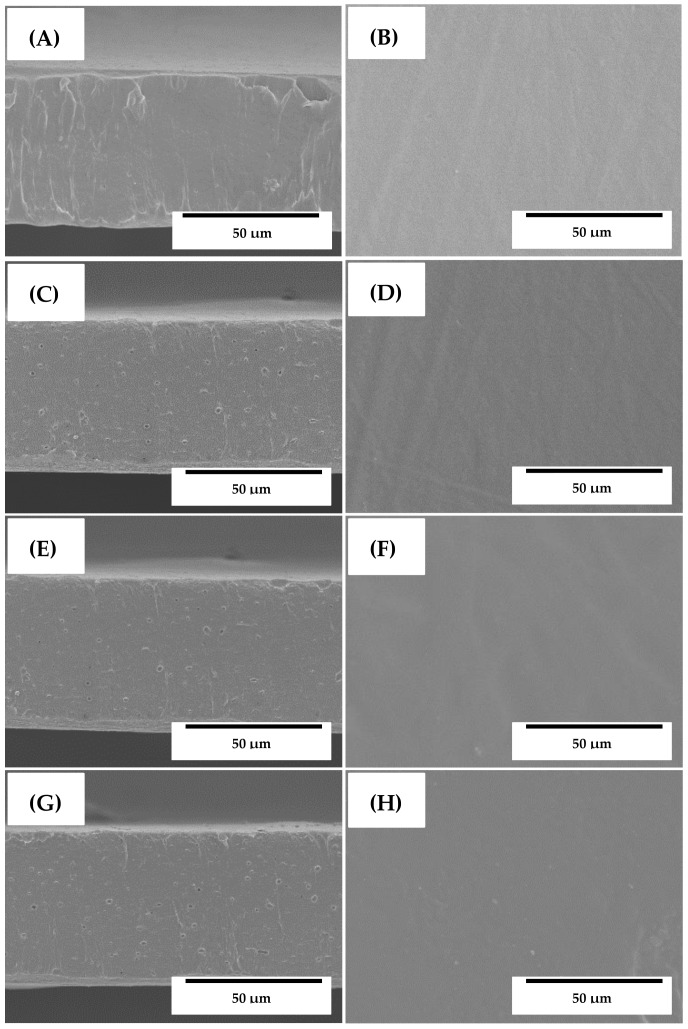
Scanning electron microscopy (SEM) micrographs of the electrospun films in their cross-section (left column) and top view (right column) of: (**A**,**B**) Neat poly(3-hydroxybutyrate-*co*-3-hydroxyvalerate) (PHBV); (**C**,**D**) Oregano essential oil (OEO)-containing PHBV; (**E**,**F**) Rosemary extract (RE)-containing PHBV; (**G**,**H**) Green tea tree extract (GTE)-containing PHBV. Scale markers of 50 μm.

Figure 2 shows the SEM images of the electrospun materials, after annealing at 125 °C, in their cross-section and top views. In all cases, one can observe that the thermal post-treatment on the electrospun mats resulted in the formation of a continuous film. Figure 2A corresponds to the cross-section of the neat PHBV film, that is, without OEO and NEs, which presented an average thickness of ~80 μm. In Figure 2B, one can observe that the film sample also exhibited a homogeneous surface without cracks and/or pores. Similar morphologies were reported, for instance, by Cherpinski et al. [38] for electrospun PHB fibers thermally post-treated at 160 °C. The particular change from fiber-based to film-like morphology was ascribed to a process of fibers coalescence during annealing. Figure 2C,E,G show the cross-sections of the electrospun PHBV films containing OEO, RE, and GTE, respectively. The thicknesses of all the film samples were kept at ~80 μm. The presence of a certain number of pores can be related to the partial evaporation of the oily materials enclosed in the PHBV film during the thermal post-treatment. Similar voids were observed in the electrospun PHBV films derived from biowaste by Melendez-Rodriguez et al. [39], when temperatures close to *T_m_* were applied, which was ascribed to the partial material melting and/or degradation. However, in Figure 2D,F,H, showing the top view of the film samples containing the active substances, it can be seen that the PHBV films still showed a smooth and homogeneous surface without pores and cracks. Therefore, despite the fact that the active substances were partially released during the film-forming process, a good compatibility and then a high solubility of OEO and the NEs with the PHBV matrix was attained.

### 3.3. Optical Properties

**Figure 3:**
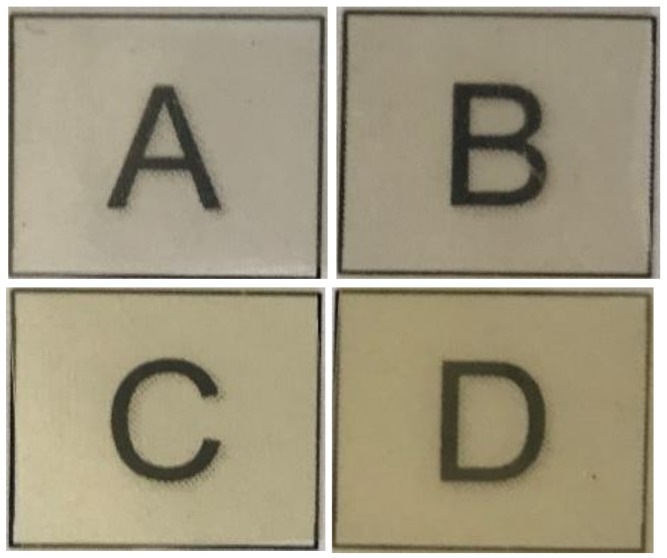
Visual aspect of the electrospun films of: (**A**) Neat poly(3-hydroxybutyrate-*co*-3-hydroxyvalerate) (PHBV); (**B**) Oregano essential oil (OEO)-containing PHBV; (**C**) Rosemary extract (RE)-containing PHBV; (**D**) Green tea tree extract (GTE)-containing PHBV. Films are 1.5 × 1.5 cm^2^.

Figure 3 shows the visual aspect of the electrospun PHBV films to evaluate their contact transparency. The effects of the addition of OEO and the NEs on the color coordinates (L*, a*, b*) and the values of Δ*E*, T, and O of the electrospun PHBV films are shown in Table 2. One can observe that all the here-prepared PHBV films presented a high contact transparency, but they also developed a slightly yellow appearance when the active substances were incorporated. The Δ*E* values of the active PHBV films with respect to the neat PHBV film were 8.36, 7.52, and 15.82 for the films with OEO, RE, and GTE, respectively. Therefore, the highest color change was observed for the GTE-containing PHBV film. The main changes observed were based on a decrease in brightness (L*) and an increase in the b* coordinate, that is, a yellower material, which was related to the intrinsic color of the added active substances.

One can also observe that the OEO-containing PHBV film presented a transparency similar to that of the neat PHBV film, both having T values in the range of 3–4, which indicated a greater passage of visible light through the material. However, the incorporation of RE and, particularly, of GTE resulted in an increase of T up to values of 6.4 and 16.4, respectively. Therefore, the capacity of transmission of visible and UV light of the films was significantly reduced by the addition of RE and GTE (*p* < 0.05), causing a phenomenon of light scattering due to the characteristic tones of the active substances. Similarly, whereas opacity was kept relatively low for the neat PHBV film and the OEO-containing PHBV films, which both had O values in the 0.015–0.02 range, these values increased up to 0.026 and 0.067 for the RE- and GTE-containing PHBV films, respectively. Then, the presence of the latter active substances, particularly GTE, reduced the transparency properties by blocking the passage of UV-Vis light and it increased the opacity of the films, caused by the scattering of light. However, as other authors have previously stated, this property can be also a desired characteristic in some packaging materials for the protection of foodstuff from light, especially UV radiation, which can cause lipid oxidation in the food products [1,40]. In this sense, the work reported by Gómez-Estaca et al. [52] also concluded that the addition of certain NEs to fish gelatin films decreased the transparency of the films and increased the opacity of the final material.

### 3.4. Thermal Stability

**Figure 4:**
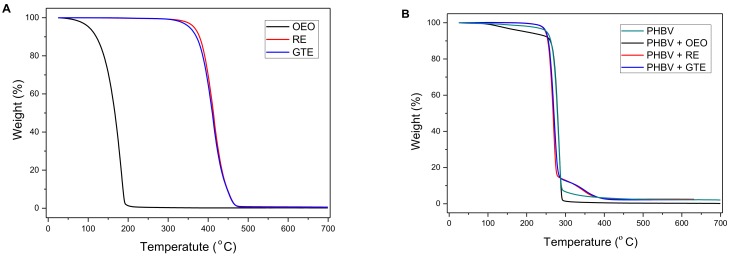
Weight loss as a function of temperature for: (**A**) Oregano essential oil (OEO), rosemary extract (RE), and green tea tree extract (GTE); (**B**) Electrospun films of neat poly(3-hydroxybutyrate-co-3-hydroxyvalerate) (PHBV) and PHBV containing OEO, RE, and GTE.

Figure 4 includes the weight loss curves of the free active substances and of the electrospun PHBV films obtained by TGA. The curves for the neat OEO, RE, and GTE are shown in Figure 4A, while the values of the onset degradation temperature, that is, the temperature at 5% weight loss (*T_5%_*), degradation temperature (*T_deg_*), and residual mass at 700 °C are gathered in Table 3. One can observe that OEO presented the lowest thermal stability, showing values of *T_5%_* and *T_deg_* of 101.5 °C and 178.4 °C, respectively, with a respective weight loss of 74.16% at *T_deg_*, corresponding to the volatilization and/or degradation of low-M_W_ volatile compounds present in the OEO (e.g., carvacrol, thymol, and pinene). In this sense, other authors have also reported that the EOs and NEs of oregano are among the most thermally unstable active substances. For instance, Barbieri et al. [53] reported that 96–97% of the OEO’s weight was lost between 200 °C and 216 °C, attributed to its volatilization. In another work, Yang et al. [54] determined a significant degradation of all terpenes extracted oregano leaves in the 200–250 °C range. Similarly, Gibara Guimarães et al. [55] reported the fully thermal decomposition of carvacrol, the most representative active compound of oregano, at 168 °C. Opposite to OEO, both RE and GTE showed a high thermal stability with a similar mass loss profile. In particular, both active substances presented *T_5%_* values over 350 °C, with *T_deg_* values of 412.7 °C (52.45%) and 411.5 °C (49.89%) for RE and and GTE, respectively. Similar results, though slighlty lower, were reported for RE by Piñeros-Hernandez et al. [56], showing a significant mass loss at 300 °C, corresponding to the decomposition of phenolic diterpenes, that is, carnosic acid, carnosol, and rosmarinic acid. Likewise, Cordeiro et al. [57] obtained a mass loss as low as 6% up to 190 °C. In the case of GTE, López de Dicastillo et al. [58] determined that it remained stable up to the range of 200–400 °C, where the thermal degradation of partially glycosylated catechins occurs. Furthermore, all active substances produced a residual mass below 1%. 

In Figure 4B, the weight loss curves of the electrospun PHBV films containing OEO, RE, and GTE are gathered. The neat PHBV film was thermally stable up to 251.5 °C, showing a *T_deg_* value of 278.7 °C (47.74%) and a residual mass of 2.10%. While the incorporation of RE and GTE slightly reduced the thermal stability by 5–10 °C, the presence of OEO considerably reduced the onset of degradation, showing a *T_5%_* value of 197.5 °C. It is also worthy to mention, however, that all active substances increased the thermally decomposed mass at *T_deg_*, that is, the weight values decreased to the 60–70 % range. Therefore, the here-produced active PHBV films were stable up to 200 °C, which certainly opened up their application as an active food packaging interlayer and/or coating. 

### 3.5. Water Contact Angle

**Figure 5:**
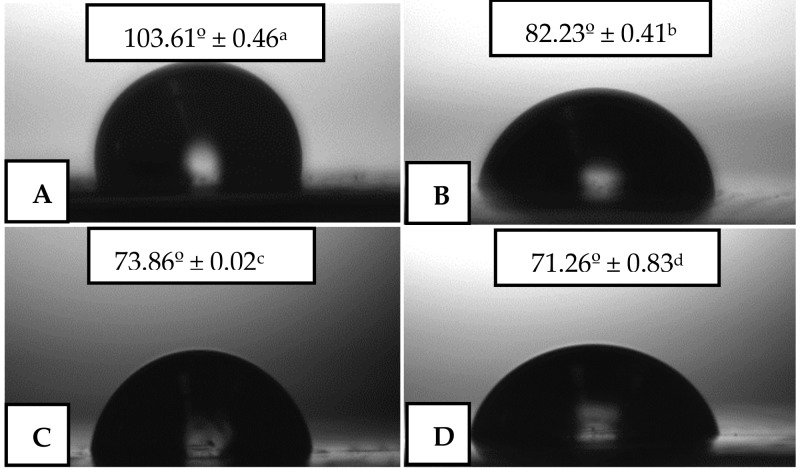
Water contact angle of the electrospun films of: (**A**) Neat poly(3-hydroxybutyrate-*co*-3-hydroxyvalerate) (PHBV); (**B**) Oregano essential oil (OEO)-containing PHBV; (**C**) Rosemary extract (RE)-containing PHBV; (**D**) Green tea tree extract (GTE)-containing PHBV.

The water contact angle refers to the degree of affinity of water with a surface, which defines the degree of hydrophilicity/hydrophobicity of a given polymer material [59]. In Figure 5, the water drop images on the films, as well as the values of their contact angles, are shown for the electrospun PHBV films. In Figure 5A, one can observe that the neat PHBV film presented an angle of 103.61°, which is characteristic of hydrophobic materials [60]. In all cases, the incorporation of the active substances resulted in a significant decrease in hydrophobicity (*p* < 0.05). Figure 5B shows that the OEO-containing PHBV film presented a water contact angle of 82.23°, whilst these values were even lower for both the films containing RE (Figure 5C), that is, 73.86°, and GTE (Figure 5D), that is, 71.26°. The reduction achieved could be related to the presence of the oily molecules on the surfaces of the PHBV films, which decreased the surface tension. A similar decrease in the water contact angles was observed by Galus and Kadzińska [61] in whey protein isolate (WPI) edible films when almond and walnut oils were added. In any case, following the terms "hydrophobic" and "hydrophilic", defined for θ > 65 ° and ≤65 °, respectively [62], the angles for each of the films studied were still within the hydrophobic range.

### 3.6. Active Properties

#### 3.6.1. Antimicrobial Activity

The EOs and NEs obtained from aromatic plants are constituted by a wide range of active compounds that are responsible for antimicrobial and antioxidant activity, which has promoted their application in active food packaging [1,41,42]. Table 4 shows the MIC and MBC values of the neat OEO, RE, and GTE against strains of *S. aureus* (Gram positive, G+) and *E. coli* (Gram negative, G-). The results showed that the OEO presented the highest antibacterial effect against both bacterial strains, having achieved identical MIC and MBC values, that is, 0.625 μL/mL, against *E. coli*, and 0.312 μL/mL, against *S. aureus*. The fact that the MIC and MBC values were identical can be related to the high effectivity of the natural compounds that were achieved, at the same time, the inhibition of microbial growth and the elimination of 99.9% of the microorganisms [63]. The antimicrobial activity of the OEO has been mainly ascribed to its high content in carvacrol and thymol [64,65]. RE presented MIC values of 10 μL/mL and 5 μL/mL against *E. coli* and *S. aureus*, respectively, whereas its MBC values were 20 μL/mL, against *E. coli*, and 10 μL/mL, against *S. aureus*. The main compounds responsible for the antimicrobial activity of RE were ∝-pinene, myrcene, camphor, 1,8-cineole, and camphene [29,66,67]. Likewise, GTE showed the lowest antimicrobial performance. This NE presented MIC values of 160 µL/mL, against *E. coli*, and 80 µL/mL, against *S. aureus*. Its MBC values were 160 µL/mL, against *E. coli*, and 40 µL/mL, against *S. aureus.* Gallic acid (GA), theobromine, chlorogenic acid, and caffeic acid are known to be responsible for its antimicrobial activity [32,68]. Most research related to MIC and MBC determination has been conducted with these EOs and NEs, finding that these compounds have a broad inhibition spectrum against G+ bacteria, but they are not as efficient against some G- bacteria [69,70]. The values of the MBC and MIC for OEO were the same, while for RE and GTE, the MBC values were higher than the MIC values. This fact is related to the effectiveness of the active compounds, the susceptibility of the microorganisms, and the variation in the penetration rate of the extracts through the cell wall and the structures of the cell membrane [71,72].

The antimicrobial properties of the electrospun PHBV films containing OEO, RE, and GTE were also evaluated using the JIS Z2801 against *S. aureus* and *E. coli* bacteria, in both an open and closed system, for 1, 8, and 15 days. In relation to the open system, as shown in Table 5, one can observe that the films containing OEO showed the strongest inhibition. These films provided a strong reduction (*R* ≥ 3), that is, with a reduction of 3 log units, against *S. aureus,* and also a high antimicrobial effect, though slightly lower, presenting *R* values of 2.7–2.9 against *E. coli*. In the case of the films containing RE and GTE, the films yielded a bacteriostatic effect (1 ≤ R < 3) against both bacteria. The antimicrobial effect of RE was also approximately 1 log units higher than that observed for GTE. These results agreed with the MIC and MBC described above, where OEO inhibited the growth of *E. coli* and *S. aureus* at lower contents, whilst RE and GTE showed higher MIC and MBC values. It is also worthy to mention that in all cases, the bacterial reduction slightly increased over the days, which can be related to the slow release of the active compounds to the surface of the films. In comparison to the previous results of electrospun antimicrobial films reported by Jeong-Ann Parka and Song-Bae Kim [73] in open systems, it was observed that the inhibition of *S. aureus* increased from a 0.6 log of reduction, at the initial time, to a 1.2 log of reduction, after 120 min. In another study, Figueroa et al. [42] also reported an increase in the bacterial inhibition with the passage of storage days, showing a 3.9 log of reduction after 10 days against *S. aureus*.

As shown in Table 6, for all the samples, the reduction was slightly higher in the closed system than in the open one. While the films containing OEO presented the strongest inhibition (*R* ≥ 3) after 15 days of storage in the closed system against the two bacterial strains, the films that contained RE and GTE showed a significant inhibition (1 ≤ R < 3). This result could be attributed to the accumulation of volatile active compounds in the headspace of the closed chamber. There are a limited number of studies reporting the antimicrobial performance of the active films in closed systems, which indeed are more practical from the point of view of packaging and the design of containers to avoid deterioration of food products during storage. For instance, Torres-Giner et al. [74] developed a multilayer system, based on an electrospun coating of zein composite nanofibers containing thymol on a polylactide (PLA) film, that was evaluated against *Listeria monocytogenes* in a closed atmosphere in desiccators. It was reported that a concentration as low as 1.6 ppm was able to produce a decrease in the CFU of about 3 log units, whereas above 6.1 ppm, no CFU were detected. The high antimicrobial performance achieved was ascribed to the capacity of the electrospun material to release the bioactive in a sustained manner. The results are of potential interest in packaging applications, since the antimicrobial effect was not only successfully achieved in open packaging systems, but it also prolonged and improved over time in closed packaging systems, thereby extending the shelf life of perishable foods [75,76].

#### 3.6.2. Antioxidant Activity

The antioxidant activity of the EOs and NEs, obtained from aromatic plants, is conferred by their phenolic compounds. The DPPH free radical method is an antioxidant assay based on an electron-transfer that produces a violet solution in methanol. This free radical, stable at room temperature, is reduced in the presence of an antioxidant molecule (active compound), giving rise to a colorless solution [77]. The use of the DPPH assay provides an easy and rapid way to evaluate antioxidants using spectrophotometry. In the conservation of foods, substances with character antioxidants are of great interest because the main cause of food deterioration results from enzymatic reactions that trigger the oxidation of lipids and carbohydrates [78,79].

The percent inhibition and the equivalent concentration in micrograms of trolox per gram of sample of the neat OEO, RE, GTE, and of the electrospun fibers and films of the PHBV containing the active substances are shown in Table 7. One can observe that all the EOs and NEs showed DPPH radical scavenging activity. OEO presented the highest percentage of inhibition (91.96%) attributed to its main active compounds (e.g., carvacrol, thymol, p-cymene, γ-terpinene) [64]. Similarly, Chun et al. [80] reported an inhibition percentage of DPPH of 82% for oregano extracts. RE presented a percentage of inhibition of 75.24%, which was in agreement with, for instance, Bajalan et al. [81] who reported an inhibition percentage of DPPH of 73.69%. GTE showed an inhibition of 71.77%, conferred by the relative amount of catechins and GA [82]. Afroz Bakht et al. [83] studied the antioxidant activity from DPPH of five commercial teas, finding percentages of inhibition in the 24–71% range. In addition, Lu and Chen [84] reported percentages of inhibition between 33% and 62%. In this sense, it is important to highlight that antioxidant activity is dependent on the quantity of secondary metabolites that the plant manages to synthesize in its development stage, which is influenced by the variety of the plant, the environmental conditions [85,86,87], and the extraction method used [83].

One can observe that the antioxidant activity decreased in the electrospun PHBV fibers containing OEO, RE, and GTE. After the electrospinning process, the fibers with OEO showed a percentage of inhibition of 43.14%, whereas the fibers containing RE and GTE showed values of 25.82% and 22.12%, respectively. In all cases, there was a decrease in the antioxidant activity of between 20% and 30% compared to the pure OEO, RE, and GTE. The lower antioxidant inhibition of the active compounds inside the fibers could be related to polarity differences between the solvent and the polymer, the stirring process applied to the active solution prior to electrospinning, and the loss of volatiles compounds during the electrospinning process [44].

Table 8 shows the percentages of inhibition and the equivalent concentration in micrograms of trolox per gram of sample of the electrospun PHBV films containing OEO, RE, and GTE, evaluated in the so-called open and closed systems on days 1, 8, and 15. Films containing OEO exhibited the highest inhibition of DPPH at day 1 (24.54%), followed by films with the RE (15.59%) and films with the GTE (11.14%). Over time, all the films decreased their antioxidant activity, obtaining no significant differences in the results between each storage system. Thus, after 15 days, an inhibition percentage in the range of 8.83–10.55% was obtained for the films containing OEO. At this time, the inhibition percentage ranges for the films containing RE and GTE were 6.91–7.31% and 5.45–6.68%, respectively. As can be observed, the antioxidant activity decreased when the EOs and NEs were included in the PHBV fibers and, more intensively, when the films were formed. Likewise, during the days of storage, a continuous release of the characteristic volatile compounds of each EOs and NEs was produced, which was evidenced by the low percentage of DPPH inhibition at day 15 for all the samples. Previous reports have indicated that the degree of antioxidant power of biodegradable films is generally proportional to the amount of added antioxidant additives, while the thermal process for obtaining the films also affects the bioactivity, since most of the bioactive compounds are sensitive to temperatures above 80 °C [47,88]. Regardless of this, all the films presented antioxidant performance and they can, therefore, be applied in antioxidant active packaging systems to extend the shelf life of packaged food products, thus minimizing the development of off-flavors, color and flavor changes, and nutritional losses [89,90]. 

## 4. Conclusions

The evaluation of the active properties, that is, antimicrobial and antioxidant properties, of OEO, RE, and GTE showed that OEO was the active substance that presented the highest antimicrobial activity against *S. aureus* and *E. coli*. This effect was mainly attributed to the effectiveness of its most representative active compounds, that is, carvacrol and thymol, showing identical MIC and MBC values of 0.312 and 0.625 µL/mL, respectively. The antioxidant activity of OEO was also higher than that for the RE and GTE. In particular, the percentage of inhibition of the DPPH was 91.96%. Thereafter, these active substances were incorporated at 10 wt.% into fruit waste derived PHBV by electrospinning. To this end, the solution properties of the PHBV containing OEO, RE, and GTE were first evaluated to determine the optimal conditions to obtain homogenous fibers. The diameters of the fibers were relatively similar, with a mean size of approximately ~0.8 μm, being uniform and smooth, without any superficial and structural defects. It was observed that the addition of the OEO and the NEs did not alter the fiber formation during electrospinning or the morphology of the electrospun ultrathin PHBV fibers. A good compatibility and, then, high solubility of the OEO and NEs with the PHBV matrix was considered.

In order to obtain an interesting active continuous layer to be applied in the design of biopackaging, the electrospun mats of the PHBV fibers containing the active substances were subjected to a thermal post-treatment at 125 °C. Continuous PHBV films of ~80 μm, with a smooth surface were obtained, though the presence of the active substances induced a slight porosity in their cross-section. The optical properties of the PHBV films were slightly impaired by the addition of the active substances, particularly GTE, reducing their transparency from 3.13 up to 16.42 through blocking the passage of UV-Vis light and increasing their opacity, which was caused by the scattering of light. In any case, all the PHBV films were contact transparent. All active substances also decreased the values of the *T_onset_* by 54 °C for OEO, 3 °C for RE, and 2.2 °C for GTE, and the thermally decomposed mass at *T_deg_* decreased to the 60–70% range. However, all the active PHBV films were stable up to 200 °C. Referring to the hydrophobicity of the films, the addition of active substances decreased the superficial tension of the PHBV films with respect to the control, but the angles for each of the films studied were still within the hydrophobic range. The PHBV films containing OEO, RE, and GTE showed antimicrobial activity against strains of *S. aureus* and *E. coli* in both the here-studied open and closed systems, where the bacterial reduction improved over time due to the release and accumulation of the active compounds on the film surface. The antimicrobial activity was higher in the case of the closed system due to the presence of volatiles stored in the headspace. The films containing OEO presented the highest reduction values against the two bacterial strains (*R* ≥ 3), while the films containing RE and GTE showed lower reduction values (1 ≤ *R* < 3), which agreed with the MIC and MBC values of the pure active substances. The antioxidant activity of the fibers and films was much lower than that of the neat active substances, which was related to entrapment and loss during electrospinning and film processing, and which was also reduced with the passage of days due to the continuous release of the active compounds. 

The here-developed electrospun PHBV layers with OEO, RE, and GTE are potential candidates for use in the design of sustainable active multilayer biopackaging. The antimicrobial and antioxidant performance of these materials is advantageous to prolonging the shelf life of foods, delaying the proliferation of microorganisms, and the enzymatic oxidation of foodstuffs.

## Figures and Tables

**Table 1:** Solution properties of poly(3-hydroxybutyrate-*co*-3-hydroxyvalerate) (PHBV) containing oregano essential oil (OEO), rosemary extract (RE), and green tea tree extract (GTE).

Samples	Apparent Viscosity (cP)	Surface Tension (mN/m)	Conductivity (µs)
PHBV	212.4 ± 0.04 ^a^	25.3 ± 0.05 ^a^	0.40 ± 0.01 ^a^
PHBV + OEO	205.5 ± 0.01 ^b^	25.5 ± 0.07 ^a^	0.42 ± 0.02 ^a^
PHBV + RE	208.1 ± 0.03 ^c^	25.4 ± 0.09 ^a^	0.41 ± 0.01 ^a^
PHBV + GTE	206.9 ± 0.05 ^d^	25.5 ± 0.06 ^a^	0.39 ± 0.01 ^a^

^a–d^ Different letters in the same column indicate a significant difference (*p* < 0.05).

**Table 2:** Color parameters (ΔE*, a*, b*, and L*) and transparency characteristics of the electrospun films of poly(3-hydroxybutyrate-*co*-3-hydroxyvalerate) (PHBV) containing oregano essential oil (OEO), rosemary extract (RE), and green tea tree extract (GTE).

Samples	a*	b*	L*	ΔE*	T	O
**PHBV**	0.87 ± 0.07 ^a^	−0.38 ± 0.02 ^a^	89.82 ± 0.06 ^a^	-	3.13 ± 0.02 ^a^	0.016 ± 0.06 ^a^
**PHBV + OEO**	1.13 ± 0.05 ^b^	6.67 ± 0.03 ^b^	85.35 ± 0.07 ^b^	8.36 ± 0.08 ^a^	3.55 ± 0.03 ^b^	0.019 ± 0.08 ^b^
**PHBV + RE**	0.04 ± 0.01 ^c^	6.67 ± 0.08 ^b^	87.33 ± 0.01 ^c^	7.52 ± 0.06 ^b^	6.44 ± 0.02 ^c^	0.026 ± 0.05 ^c^
**PHBV + GTE**	0.07 ± 0.09 ^c^	14.45± 0.05 ^c^	84.38 ± 0.03 ^d^	15.82 ± 0.05 ^c^	16.42 ± 0.06 ^d^	0.067 ± 0.04 ^d^

a*: red/green coordinates (+a red, −a green); b*: yellow/blue coordinates (+b yellow, −b blue); L*: Luminosity (+L luminous, −L dark); ΔE*: color differences; T: transparency; O: opacity. ^a–d^ Different letters in the same column indicate a significant difference (*p* < 0.05).

**Table 3:** Thermal properties of oregano essential oil (OEO), rosemary extract (RE), and green tea tree extract (GTE) and of the electrospun films of poly(3-hydroxybutyrate-*co*-3-hydroxyvalerate) (PHBV) containing OEO, RE, and GTE in terms of temperature at 5 % weight loss (*T_5%_*), degradation temperature (*T_deg_*), and residual mass at 700 °C.

Sample	*T_5%_* (°C)	*T_deg_* (°C)	Mass Loss (%)	Residual Mass (%)
OEO	101.5	178.4	74.16	0.14
RE	364.0	412.7	52.45	0.48
GTE	352.7	411.5	49.89	0.56
PHBV	251.5	278.7	47.74	2.10
PHBV + OEO	197.5	283.6	69.58	0.16
PHBV + RE	248.5	270.8	60.94	2.49
PHBV + GTE	249.3	273.8	61.65	2.21

**Table 4:** Minimum inhibitory concentration (MIC) and minimum bactericidal concentration (MBC) of oregano essential oil (OEO), rosemary extract (RE), and green tea tree extract (GTE) against *S. aureus* and *E. coli*.

Sample	Bacteria	MIC	MBC
OEO	*E. coli*	0.625 µL/mL	0.625 µL/mL
	*S. aureus*	0.312 µL/mL	0.312 µL/mL
RE	*E. coli*	10 µL/mL	20 µL/mL
	*S. aureus*	5 µL/mL	10 µL/mL
GTE	*E. coli*	160 µL/mL	160 µL/mL
	*S. aureus*	40 µL/mL	80 µL/mL

**Table 5:** Antibacterial activity against *S. aureus* and *E. coli* of the electrospun poly(3-hydroxybutyrate-*co*-3-hydroxyvalerate) (PHBV) films containing oregano essential oil (OEO), rosemary extract (RE), and green tea tree extract (GTE) in the open system for up to 15 days.

Microorganism		Day	Control Sample log (CFU/mL)	Test Sample log (CFU/mL)	*R*
***S. aureus***	PHBV + OEO	1	6.91 ± 0.06	3.78 ± 0.08	3.13 ± 0.06
		8	6.88 ± 0.50	3.68 ± 0.03	3.20 ± 0.04
		15	6.89 ± 0.20	3.65 ± 0.10	3.24 ± 0.15
	PHBV + RE	1	6.87 ± 0.03	4.07 ± 0.07	2.80 ± 0.06
		8	6.88 ± 0.09	4.01 ± 0.03	2.87 ± 0.03
		15	6.87 ± 0.02	3.95 ± 0.01	2.92 ± 0.02
	PHBV + GTE	1	6.91 ± 0.10	5.00 ± 0.32	1.91 ± 0.20
		8	6.89 ± 0.23	4.94 ± 0.18	1.95 ± 0.13
		15	6.92 ± 0.11	4.93 ± 0.22	1.99 ± 0.19
***E. coli***	PHBV + OEO	1	6.95 ± 0.30	4.24 ± 0.09	2.71 ± 0.10
		8	6.90 ± 0.08	4.09 ± 0.10	2.81 ± 0.20
		15	6.87 ± 0.07	4.01 ± 0.03	2.86 ± 0.05
	PHBV + RE	1	6.89 ± 0.03	5.00 ± 0.06	1.89 ± 0.05
		8	6.90 ± 0.09	4.96 ± 0.07	1.94 ± 0.07
		15	6.88 ± 0.08	4.91 ± 0.09	1.97 ± 0.07
	PHBV + GTE	1	6.89 ± 0.15	5.70 ± 0.19	1.19 ± 0.17
		8	6.87 ± 0.33	5.63 ± 0.21	1.24 ± 0.23
		15	6.90 ± 0.46	5.62 ± 0.27	1.28 ± 0.31

**Table 6:** Antibacterial activity against *S. aureus* and *E. coli* of the electrospun poly(3-hydroxybutyrate-*co*-3-hydroxyvalerate) (PHBV) films containing oregano essential oil (OEO), rosemary extract (RE), and green tea tree extract (GTE) in the closed system for up to 15 days.

Microorganism		Day	Control Sample log (CFU/mL)	Test Sample log (CFU/mL)	*R*
***S. aureus***	PHBV + OEO	1	6.91 ± 0.06	3.78 ± 0.08	3.13 ± 0.06
		8	6.93 ± 0.30	3.52 ± 0.90	3.41 ± 0.30
		15	6.92 ± 0.20	3.33 ± 0.08	3.59 ± 0.07
	PHBV + RE	1	6.87 ± 0.03	4.07 ± 0.07	2.80 ± 0.06
		8	6.89 ± 0.07	3.92 ± 0.05	2.91 ± 0.04
		15	6.88 ± 0.12	3.86 ± 0.15	3.02 ± 0.11
	PHBV + GTE	1	6.91 ± 0.10	5.00 ± 0.32	1.91 ± 0.20
		8	6.89 ± 0.15	4.89 ± 0.17	2.00 ± 0.11
		15	6.86 ± 0.20	4.78 ± 0.19	2.08 ± 0.21
***E. coli***	PHBV + OEO	1	6.95 ± 0.30	4.24 ± 0.09	2.71 ± 0.10
		8	6.90 ± 0.08	3.96 ± 0.10	2.94 ± 0.30
		15	6.92 ± 0.09	3.91 ± 0.07	3.01 ± 0.06
	PHBV + RE	1	6.89 ± 0.03	5.00 ± 0.06	1.89 ± 0.05
		8	6.87 ± 0.05	4.88 ± 0.08	1.99 ± 0.09
		15	6.88 ± 0.07	4.79 ± 0.03	2.09 ± 0.05
	PHBV + GTE	1	6.89 ± 0.15	5.70 ± 0.19	1.19 ± 0.17
		8	6.91 ± 0.11	5.62 ± 0.13	1.29 ± 0.15
		15	6.90 ± 0.28	5.53 ± 0.21	1.37 ± 0.19

**Table 7:** Inhibition percentage (%) of 2,2-diphenyl-1-picrylhydrazyl radical (DPPH) and concentration (eq. trolox/g sample) of DPPH for oregano essential oil (OEO), rosemary extract (RE), and green tea tree extract (GTE) and the electrospun fibers of poly(3-hydroxybutyrate-*co*-3-hydroxyvalerate) (PHBV) containing OEO, RE, and GTE.

Sample	Inhibition Percentage (%)	Concentration (µg eq trolox/g Sample)
OEO	91.96 ± 0.03	84.34 ± 0.03
RE	75.24 ± 0.04	62.34 ± 0.03
GTE	71.77 ± 0.08	61.95 ± 0.07
PHBV + OEO	43.14 ± 0.07	28.56 ± 0.05
PHBV + RE	25.82 ± 0.07	18.31 ± 0.05
PHBV + GTE	22.12 ± 0.06	13.14 ± 0.04

**Table 8:** Inhibition percentage (%) of 2,2-diphenyl-1-picrylhydrazyl radical (DPPH) and concentration (eq. trolox/g sample) of DPPH for the electrospun poly(3-hydroxybutyrate-*co*-3-hydroxyvalerate) (PHBV) films containing oregano essential oil (OEO), rosemary extract (RE), and green tea tree extract (GTE).

		Open System		Closed System	
Sample	Day	Inhibition Percentage (%)	Concentration (µg eq trolox/g Sample)	Inhibition Percentage (%)	Concentration (µg eq trolox/g Sample)
PHBV + OEO	1	24.54 ± 0.04	26.48 ± 0.04	24.54 ± 0.04	26.48 ± 0.04
	8	16.08 ± 0.08	16.82 ± 0.09	17.43 ± 0.04	17.57 ± 0.04
	15	14.90 ± 0.06	15.75 ± 0.06	15.24 ± 0.01	16.47 ± 0.01
PHBV + RE	1	15.59 ± 0.02	16.31 ± 0.02	15.59 ± 0.02	16.31 ± 0.02
	8	10.42 ± 0.08	10.27 ± 0.08	13.50 ± 0.01	13.97 ± 0.01
	15	7.310 ± 0.04	7.710 ± 0.04	8.200 ± 0.15	8.120 ± 0.02
PHBV + GTE	1	11.14 ± 0.04	11.79 ± 0.04	11.14 ± 0.04	11.79 ± 0.04
	8	8.910 ± 0.10	8.760 ± 0.10	9.960 ± 0.02	9.820 ± 0.02
	15	6.680 ± 0.11	6.540 ± 0.11	7.800 ± 0.02	8.250 ± 0.02
